# Emerging applications and research trends of 3D printing and bioprinting in thoracic surgery: a bibliometric and visualized analysis

**DOI:** 10.3389/fsurg.2025.1710837

**Published:** 2026-01-08

**Authors:** Demiao Kong, Jingjing Deng, Qikun Mao, Xun Zhao, Limin Ye, Liankui Han, Jianfeng Zhou, Lin Yang

**Affiliations:** 1 Department of Thoracic Surgery, Guizhou Provincial People’s Hospital, Guiyang, China; 2 Digestive Department, Guizhou Provincial People’s Hospital, Guiyang, China; 3 Department of Thoracic Surgery, West China Hospital of Sichuan University, Chengdu, Sichuan, China

**Keywords:** 3D printing, bioprinting, thoracic surgery, tissue engineering, regenerative therapy

## Abstract

**Background:**

Three-dimensional (3D) printing and bioprinting technologies have rapidly evolved into essential tools in thoracic surgery, enabling personalized anatomical modeling, implant fabrication, and tissue engineering. However, the global research landscape and thematic evolution of this field remain incompletely characterized.

**Methods:**

A comprehensive bibliometric and visualized analysis was conducted using the Web of Science Core Collection, Scopus, and PubMed databases, covering studies published between 2008 and 2024. Data visualization and network analyses were performed using CiteSpace (v6.2.4R) and VOSviewer (v1.6.18) to assess publication trends, author and institutional collaborations, co-citation patterns, and keyword evolution.

**Results:**

A total of 740 publications were identified, including 627 original articles and 113 reviews, contributed by 4,077 authors from 1,277 institutions across 71 countries. Annual publications increased steadily, peaking in 2024. China ranked first in publication volume (205 papers, 27.7%), while the United States had the highest citation impact (10,969 citations; 58.66 citations per paper). The most active journals were Journal of Thoracic Disease and Medical Physics. Keyword and co-citation analyses revealed three main research phases (1): anatomical modeling and surgical simulation (2008–2015) (2); prosthetic design and clinical application (2016–2020); and (3) tissue engineering, radiotherapy guidance, and bioprinting innovations (2021–2024). Emerging hotspots included electrospinning, volatile organic compound sensing, and tumor-specific implant customization.

**Conclusion:**

Global research on 3D printing in thoracic surgery has expanded rapidly, with a clear transition from mechanical reconstruction toward biologically functional and regenerative approaches. The integration of bioprinting with advanced imaging, artificial intelligence, and robotics holds promise for personalized, precision thoracic surgery. Continued interdisciplinary collaboration will be essential to accelerate clinical translation and regulatory approval of biofabricated constructs.

## 1 Introduction

Three-dimensional (3D) printing, also known as additive manufacturing, has emerged as a disruptive technology in modern medicine (1). By enabling the precise fabrication of complex anatomical structures, patient-specific implants, and surgical models, 3D printing is transforming the landscape of thoracic surgery (2). Its applications range from anatomical visualization, preoperative planning, and intraoperative navigation to the development of personalized prostheses, airway stents, rib or chest wall reconstructions, and bioprinted scaffolds for tissue engineering (3, 4). In particular, thoracic surgeons increasingly leverage 3D printing to improve surgical precision, reduce operative time, and enhance patient outcomes in complex procedures involving the lungs, mediastinum, trachea, and chest wall (5, 6).

Over the past decade, technological advancements in imaging modalities, printing resolution, and biomaterials have significantly expanded the scope and feasibility of 3D printing in clinical thoracic practice (7, 8). Concurrently, interdisciplinary collaboration among thoracic surgeons, bioengineers, and materials scientists has led to innovations such as bioresorbable airway splints, radiotherapy phantoms, and custom-designed implants (9, 10). Despite these promising developments, the field remains heterogeneous, and there is no consolidated understanding of its global research trends, leading contributors, institutional collaborations, or emerging thematic focuses (11, 12). Previous studies have often focused on specific devices or clinical outcomes, but a holistic, data-driven exploration of the field's evolution is still absent.

Bibliometric analysis offers a powerful tool to evaluate the structure, dynamics, and impact of scientific research. By quantitatively assessing publication outputs, citation patterns, keyword co-occurrence, and co-authorship networks, bibliometrics can uncover knowledge structures, collaboration patterns, and intellectual milestones (13). Visualization software such as CiteSpace and VOSviewer further enhances the interpretation of bibliometric data, allowing researchers to identify emerging hotspots, research clusters, and interdisciplinary linkages (14). In the context of 3D printing in thoracic surgery, a rapidly evolving and multidisciplinary domain, bibliometric methods can provide valuable strategic guidance for future clinical and translational efforts.

To address this gap, we conducted a comprehensive bibliometric and visualized analysis of publications on 3D printing in thoracic surgery, covering a 17-year span from 2008 to 2024. Using robust bibliometric tools, we analyzed the distribution of publications across countries, institutions, authors, and journals, as well as co-citation networks and keyword bursts. This study aims to provide a macroscopic view of the developmental trajectory, collaborative landscape, and thematic evolution of this field, offering insights that can inform future research, innovation, and interdisciplinary integration in thoracic surgical practice.

## 2 Materials and methods

### 2.1 Data acquisition

The Web of Science Core Collection (WoSCC), Scopus, and PubMed were selected as the data sources for this bibliometric analysis in order to ensure comprehensive coverage and minimize bias. WoSCC is widely recognized for its bibliographic accuracy and citation indexing, while Scopus and PubMed provide complementary breadth and reliability. We conducted a systematic search across these three databases to retrieve all publications related to the application of three-dimensional (3D) printing in thoracic surgery, with a publication date up to December 31, 2024. After merging the records and removing duplicates, a total of 923 unique publications were obtained. The complete Boolean search strategy used for each database was as follows: (1) Web of Science Core Collection (WoSCC): TS =((((“3D printing”) OR (“Printing, Three-Dimensional”) OR (“Three-Dimensional Printings*”) OR (“Three Dimensional Printing*”) OR (“3-D Printing*”) OR (“3-Dimensional Printing*”)) AND ((Lung* OR Pulmonary* OR “rib cage*” OR Esophag* OR Trachea* OR “chest cavity*” OR Chest*)))). (2) Scopus: TITLE-ABS-KEY ((“3D printing” OR “three-dimensional printing” OR “additive manufacturing”) AND (lung* OR pulmonary* OR rib* OR trachea* OR esophag* OR chest* OR thoracic)). (3) PubMed: (“3D printing”[MeSH Terms] OR “three-dimensional printing"[All Fields] OR “additive manufacturing”[All Fields]) AND (lung[All Fields] OR pulmonary[All Fields] OR trachea[All Fields] OR chest[All Fields] OR thoracic[All Fields]).

Inclusion criteria for eligible records were as follows: (1) studies focusing on the application of 3D printing in the field of thoracic surgery; (2) original articles or review papers published in English; and (3) published before or on December 31, 2024. Exclusion criteria included: (1) publications irrelevant to the theme of 3D printing in thoracic surgery; and (2) non-research articles such as news reports, editorials, or brief communications.

**Figure 1:**
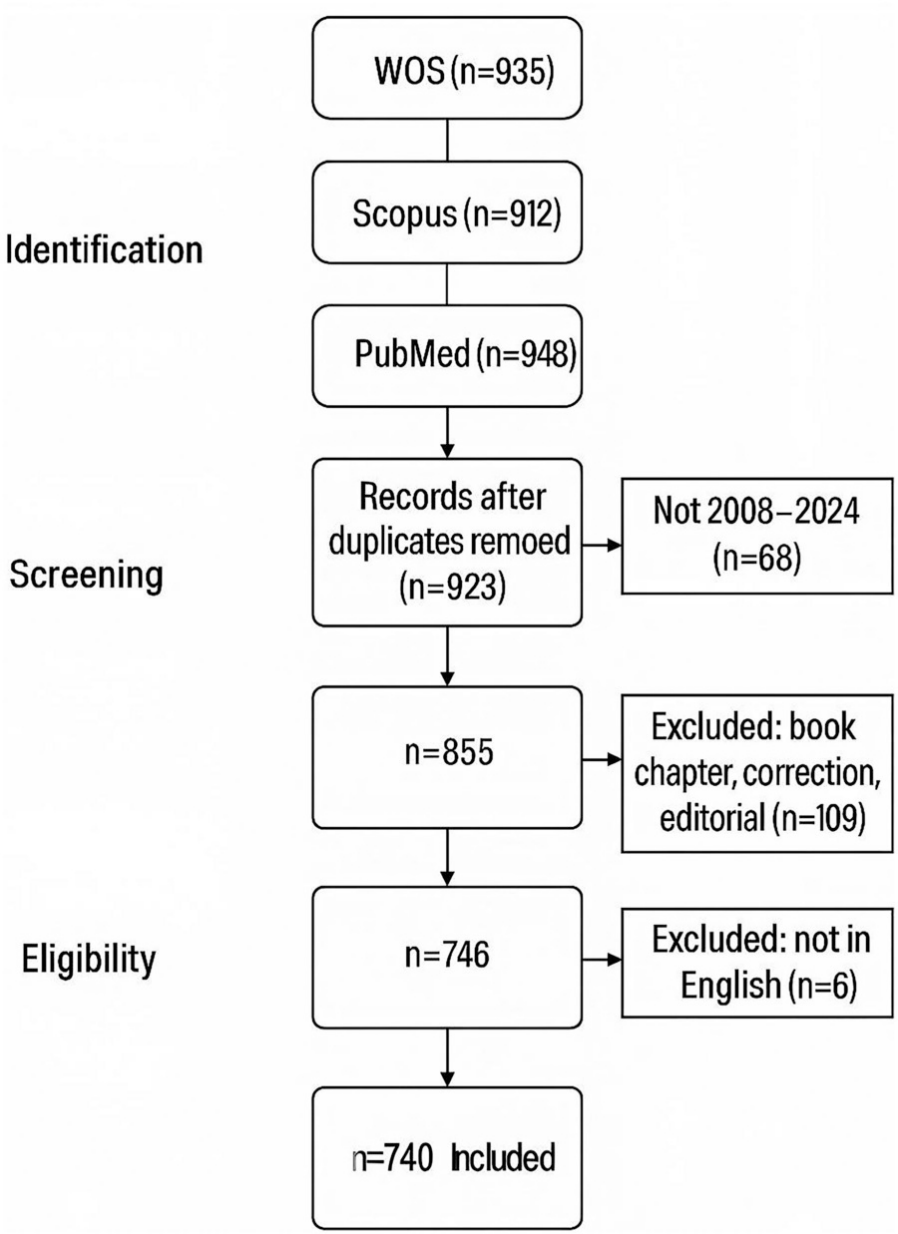
Flowchart of literature search.

To handle overlapping records retrieved from multiple databases, matching was cross-checked using DOI, article title, and first author name. Discrepancies were discussed and resolved by consensus. The final dataset included 740 unique publications for bibliometric analysis. All identified records were exported in plain text format for further analysis. The research flow chart is shown in Figure 1.

Only English-language publications were included to ensure consistency in bibliometric indexing and accurate keyword analysis across the three databases. While this approach improves data uniformity, it may introduce language and publication bias by excluding non-English studies. This limitation has been acknowledged in the Discussion section.

### 2.2 Data analysis and visualization

Descriptive statistics, including the annual publication output and the national distribution and trends of publications, were analyzed and visualized using GraphPad Prism version 8.0.2. Furthermore, CiteSpace (version 6.2.4R, 64-bit, advanced edition) and VOSviewer (version 1.6.18) were employed for advanced bibliometric network construction and knowledge visualization. VOSviewer, a Java-based software developed by Waltman et al. in 2009, enables the construction and visualization of bibliometric maps, particularly co-authorship, co-occurrence, citation, bibliographic coupling, and co-citation networks (15).

CiteSpace, developed by Professor Chaomei Chen, is a well-established tool designed to identify emerging trends and transient patterns in scientific literature through a structural and temporal analysis framework (16). The software facilitates the visualization of co-citation networks and the detection of research fronts, turning points, and intellectual base clusters. Its application in this study enables a deeper understanding of the knowledge structure, developmental trajectory, and future directions of 3D printing in thoracic surgery.

## 3 Results

### 3.1 Global trend in publication outputs and citations

A total of 740 publications related to the application of 3D printing in thoracic surgery were retrieved between January 1, 2008, and December 31, 2024. These comprised 627 original research articles and 113 review papers, involving contributions from 4,077 authors affiliated with 1,277 institutions across 71 countries and regions.

**Figure 2:**
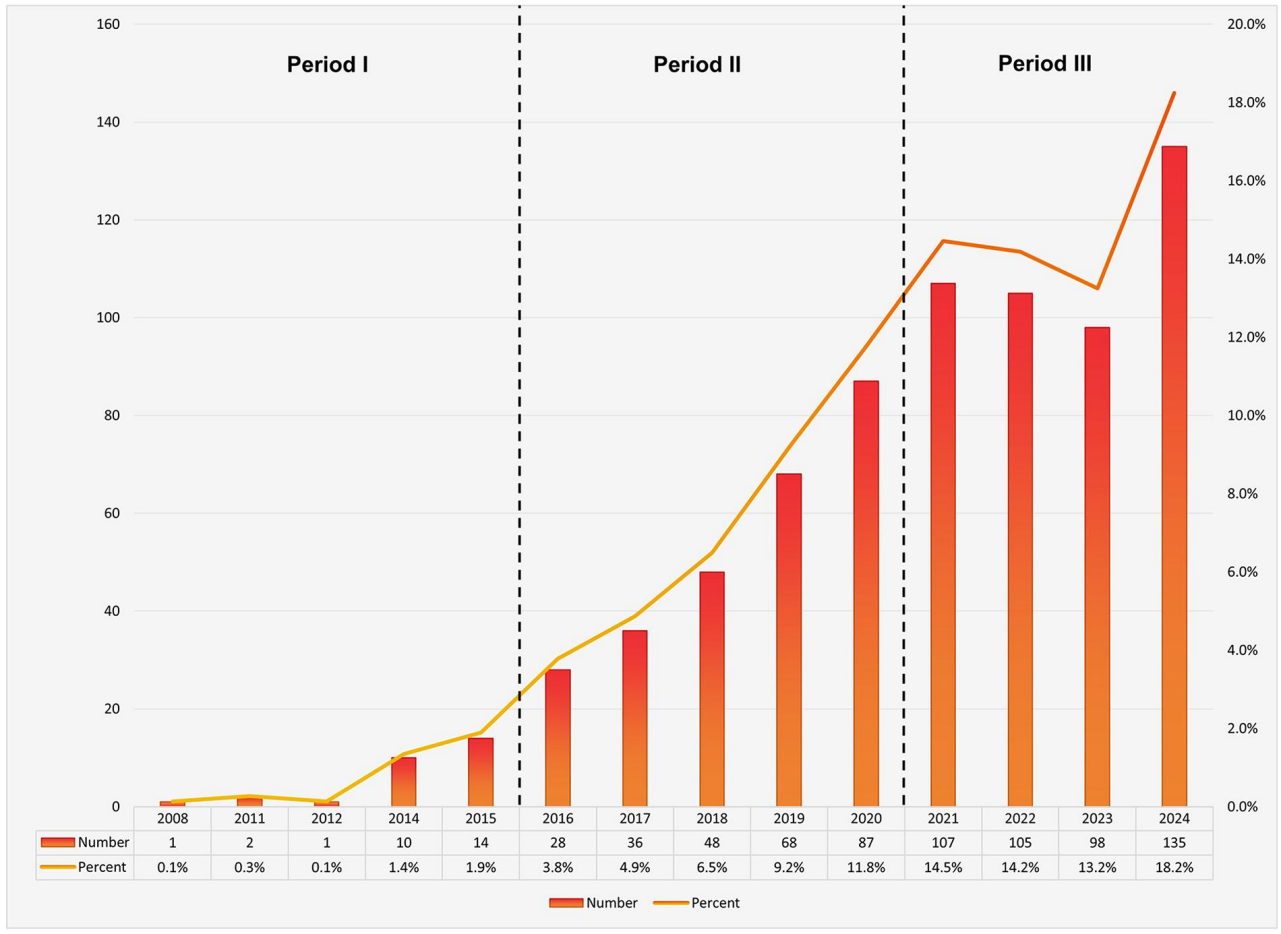
Published trend chart concerning research for 3D printing in thoracic disease.

As illustrated in Figure 2, the annual number of publications has shown a consistent upward trend since 2008. To better understand the developmental trajectory of this research field, we divided the timeline into three distinct phases. The first phase (2008–2015) was characterized by minimal scholarly attention, with fewer than 20 publications per year, suggesting limited awareness and adoption of 3D printing technologies in thoracic surgery during this early period. In the second phase (2016–2020), the annual output gradually increased, exceeding 80 publications by 2020, indicating a growing interest among researchers and the emergence of 3D printing as a relevant topic in surgical innovation. The third phase (2021–2024) marked a period of rapid expansion, with annual publication counts consistently surpassing 100, peaking in 2024. This surge reflects the significant technological advancements in 3D printing and its expanding integration into clinical and surgical applications, highlighting increasing recognition of its potential in thoracic surgery research and practice.

### 3.2 Distribution of countries/regions

**Figure 3:**
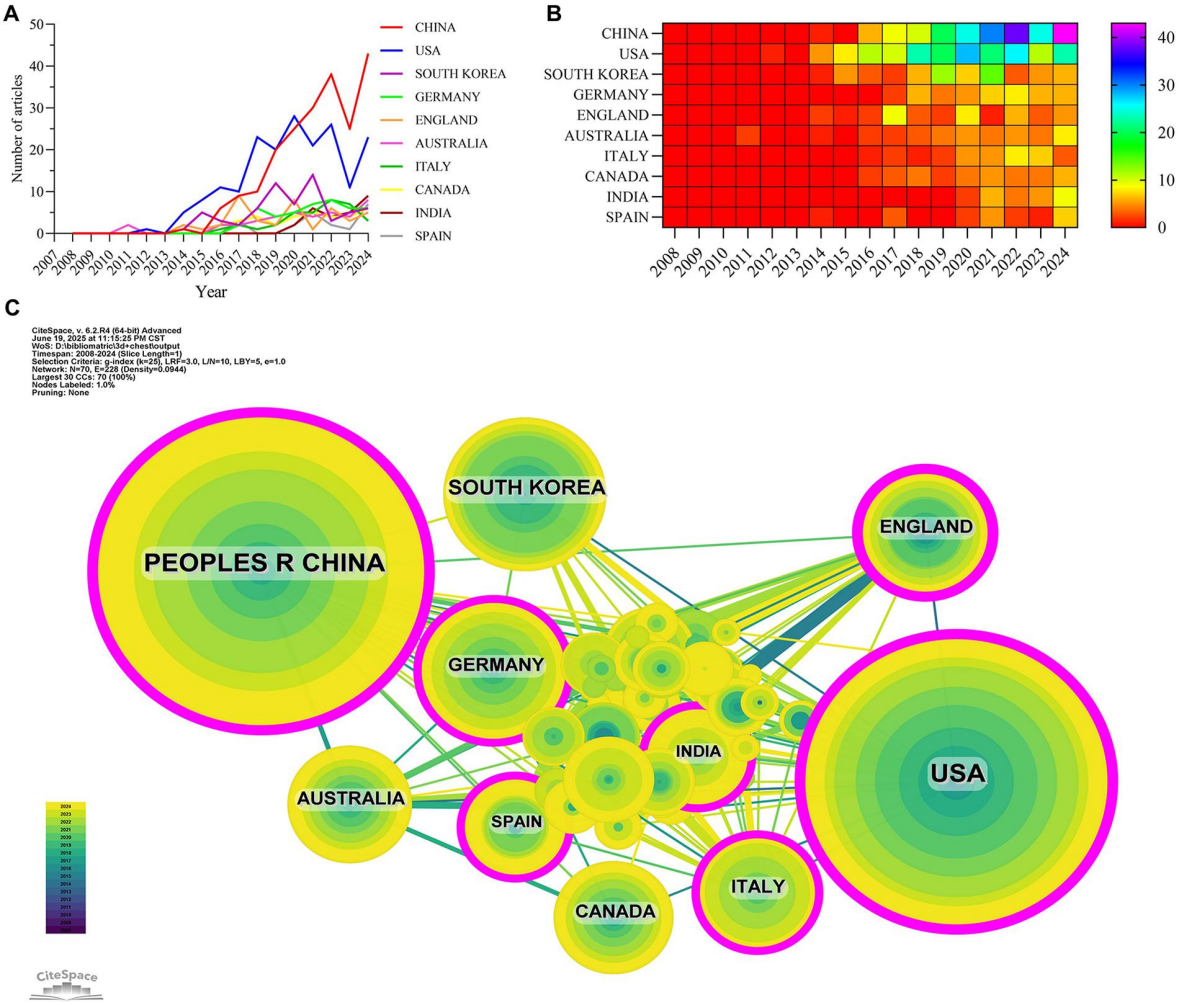
Country/region collaboration network of research of 3D printing in thoracic disease. **(A)** Line graph of national publications. **(B)** Heat map of national publications. **(C)** Networks of country cooperation, node size represents the volume of publications and the line color represents the year of collaboration.

A total of 71 countries and regions have contributed to research on the application of 3D printing in thoracic surgery. As shown in Figures 3A,B, the top 10 most productive countries over the past decade were identified based on their annual publication output. China, the United States, South Korea, Germany, and the United Kingdom ranked as the top five contributors in terms of publication volume. China led with 205 publications, accounting for 27.7% of the total output, substantially surpassing other countries in volume. Despite China's dominance in publication count, the United States exhibited a significantly higher academic influence. As shown in Table 1, articles from the U.S. accumulated 10,969 citations, far exceeding any other country. The citation-to-publication ratio for the U.S. was 58.66—the highest among all countries—indicating high citation impact and overall research quality. In contrast, although China ranked first in publication volume, it ranked second in total citations (3,023) and only ninth in citation-per-publication ratio (14.75), suggesting that the average quality or impact of its publications may be comparatively lower. This discrepancy may suggest that while China is producing a large number of publications, many of these may not yet have achieved the same level of international recognition or citation as those from the U.S. One possible explanation is that China is still in the phase of rapidly increasing research output, which may involve a greater proportion of early-stage, experimental, or less high-profile studies that take time to gain wider academic attention. The United States, on the other hand, has fewer publications but tends to focus on highly impactful, well-cited research that influences the global scientific community. This points to the maturity of research in the U.S. compared to the relatively early but growing output in China.

**Table 1:** Table of country published literature.

Rank	Country/Region	Article count	Centrality	Percentage (%)	Citation	Citation per publication
1	China	205	0.29	27.70%	3,023	14.75
2	USA	187	0.26	25.27%	10,969	58.66
3	South Korea	64	0.02	8.65%	2,292	35.81
4	Germany	44	0.16	5.95%	663	15.07
5	England	42	0.21	5.68%	1,083	25.79
6	Australia	40	0.07	5.41%	767	19.18
7	Italy	34	0.14	4.59%	950	27.94
8	Canada	34	0	4.59%	602	17.71
9	India	26	0.11	3.51%	372	14.31
10	Spain	21	0.11	2.84%	626	29.81

The international collaboration network is illustrated in Figure 3C. The United States and China, as the two most prolific countries, demonstrated close collaborative ties. The U.S. also maintained strong cooperative relationships with countries such as Italy, the United Kingdom, and India. Meanwhile, China showed notable collaboration with South Korea and Germany. Importantly, the United States not only led in citation impact but also exhibited a high betweenness centrality (0.26) in the collaboration network. This metric reflects its strategic position as a key connector and leader in the global research landscape of 3D printing in thoracic surgery. Our analysis of institutional centrality in the co-authorship network reveals a pattern of localized research efforts, particularly within countries like China, where high output is concentrated in domestic institutions. Although China has a large number of publications, the centrality of institutions in the global collaboration network is relatively low, indicating that much of the research remains isolated within national borders. In contrast, the United States, while not leading in publication volume, exhibits a much higher centrality value, suggesting that U.S. institutions are at the center of a broader, more integrated global research network. This pattern suggests that international collaboration is still limited, particularly in high-output countries like China, where there is a strong emphasis on domestic research. This finding highlights a key opportunity for improvement: increased interdisciplinary and international collaboration could accelerate the clinical translation of 3D bioprinting technologies and promote more robust global partnerships, facilitating faster innovation and cross-pollination of ideas.

### 3.3 Institutions

**Figure 4:**
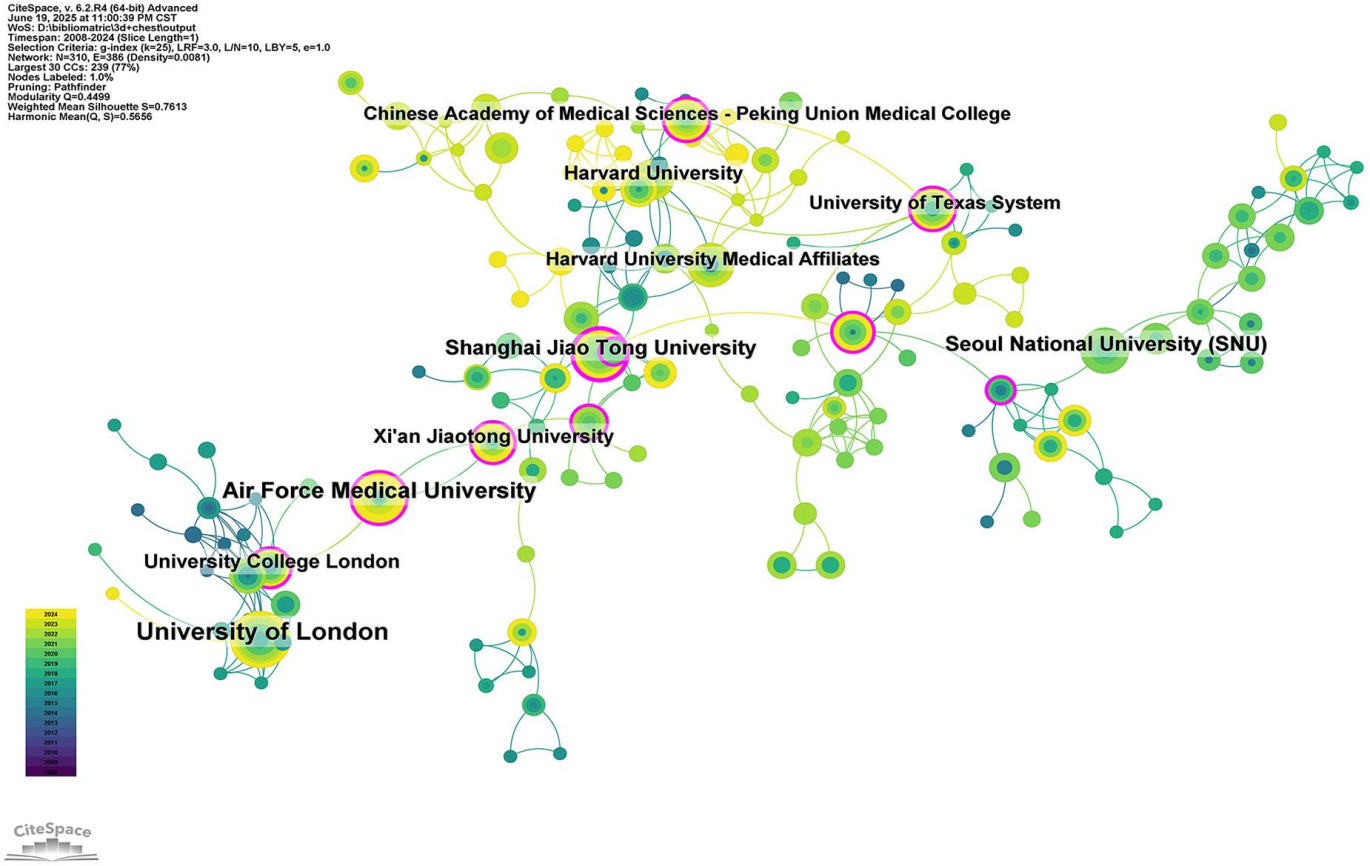
Networks of institutional co-operation.

A total of 1,277 institutions have contributed to the body of literature on 3D printing in thoracic surgery. Among the top 10 most productive institutions, five were based in the United States, three in China, and two in the United Kingdom (Table 2; Figure 4). The University of London emerged as the most prolific institution, with 25 publications garnering 309 citations, resulting in an average of 12.36 citations per article. Air Force Medical University ranked second with 19 publications and an equal total of 309 citations, yielding a higher average of 16.26 citations per publication. Shanghai Jiao Tong University followed closely, with 16 articles and 271 citations, averaging 16.94 citations per article, the highest among the top three institutions.

**Table 2:** Table of institutional published literature.

Rank	Institution	Country	Number of studies	Total citations	Average citation
1	University of London	England	25	309	12.36
2	Air Force Medical University	USA	19	309	16.26
3	Shanghai Jiao Tong University	China	16	271	16.94
4	Seoul National University (SNU)	South Korea	15	177	11.80
5	University College London	England	13	240	18.46
6	Harvard University	USA	13	417	32.08
7	Xi'an Jiaotong University	China	13	28	2.15
8	Harvard University Medical Affiliates	USA	12	187	15.58
9	Chinese Academy of Medical Sciences - Peking Union Medical College	China	11	327	29.73
10	University of Texas System	USA	11	262	23.82

Despite the high output of several leading institutions, our analysis revealed a prevalent pattern of domestic collaboration. Institutions tended to collaborate predominantly with others within the same country, with limited international institutional partnerships. This insular collaboration trend may limit the cross-pollination of innovative ideas and technological advancements.

### 3.4 Journals

**Figure 5:**
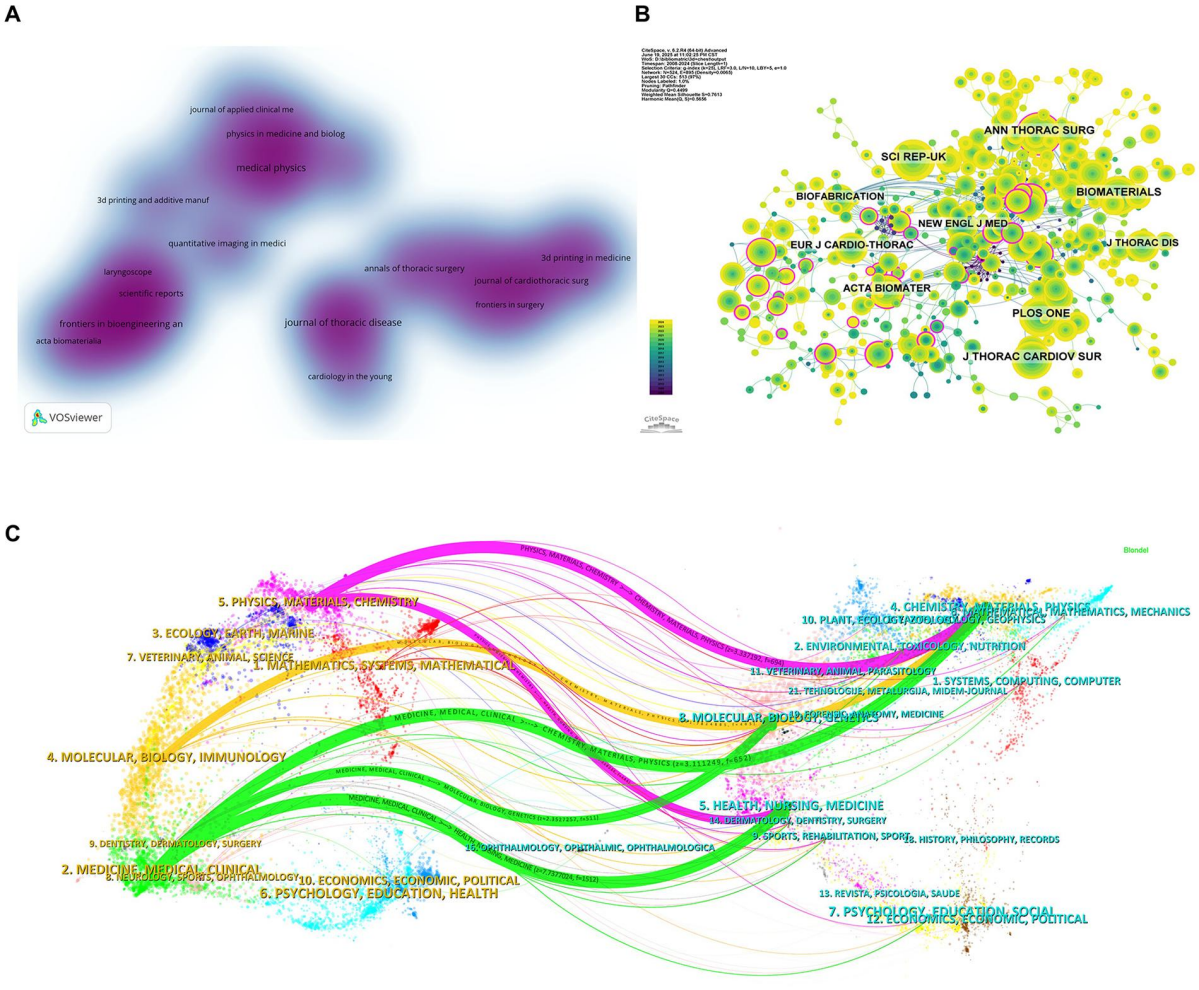
Analysis of journal sources. **(A)** Density web map of journal publications. **(B)** Co-citation network map of journals. **(C)** Dual map of journals.

Tables 3, 4 present the top 10 most productive and most co-cited journals in the field of 3D printing in thoracic surgery. The Journal of Thoracic Disease ranked first in publication volume, contributing 17 articles (2.30% of total publications), followed by Medical Physics (15 articles, 2.03%), Frontiers in Bioengineering and Biotechnology (11 articles, 1.49%), and Annals of Thoracic Surgery (9 articles, 1.22%). Among the top 10 most productive journals, Biomaterials had the highest impact factor (IF = 12.8). Notably, 80% of these journals are classified in the Q1 or Q2 quartiles of the Journal Citation Reports (JCR), indicating a generally high academic quality of publication outlets in this domain. According to the journal density visualization map, research publications in this field primarily cluster into four thematic areas: thoracic surgery, bioengineering, general surgery, and materials science (Figure 5A).

**Table 3:** Table of journal publications.

Rank	Journal	Article counts	Percentage (740)	IF	Quartile in category
1	Journal of thoracic disease	17	2.30%	2.1	Q3
2	Medical physics	15	2.03%	3.2	Q1
3	Frontiers in bioengineering and biotechnology	11	1.49%	4.3	Q1
4	Annals of thoracic surgery	9	1.22%	3.7	Q1
5	Physics in medicine and biology	9	1.22%	3.3	Q1
6	Scientific reports	9	1.22%	3.8	Q1
7	Biomaterials	8	1.08%	12.8	Q1
8	Frontiers in oncology	8	1.08%	3.5	Q2
9	Journal of cardiothoracic surgery	8	1.08%	1.5	Q3
10	3d printing in medicine	7	0.95%	3.2	Q1

**Table 4:** Co-citation table of journals.

Rank	Cited journal	Co- citation	IF(2024)	Quartile in category
1	ANN THORAC SURG	207	3.7	Q1
2	SCI REP-UK	203	3.8	Q1
3	PLOS ONE	193	2.8	Q1
4	BIOMATERIALS	191	12.8	Q1
5	J THORAC CARDIOV SUR	179	4.9	Q1
6	ACTA BIOMATER	136	9.4	Q1
7	EUR J CARDIO-THORAC	131	3.3	Q1
8	BIOFABRICATION	122	8.2	Q1
9	J THORAC DIS	118	2.1	Q3
10	NEW ENGL J MED	113	96.3	Q1

The influence of journals was further assessed by co-citation analysis, which reflects how often journals are cited together and hence their integrated impact on the scientific community. As illustrated in Figure 5B and Table 4, Annals of Thoracic Surgery was the most frequently co-cited journal (207 times), followed by Scientific Reports—UK (203 times) and PLOS ONE (193 times). Although not the most frequently cited, The New England Journal of Medicine was cited 113 times and had the highest impact factor (IF = 96.3) among the top 10 co-cited journals. Among these, 90% were classified as Q1 or Q2 journals, suggesting that foundational literature in this field is predominantly published in high-impact venues.

Further insight into the disciplinary structure and knowledge flow was obtained using the dual-map overlay of journals (Figure 5C). In this map, citing journals are shown on the left and cited journals on the right, with colored citation paths indicating cross-disciplinary citation trajectories. Six major citation pathways were identified: These citation patterns indicate a high degree of interdisciplinary integration in the field, where developments in materials science, bioengineering, and molecular biology inform clinical research and vice versa. Such knowledge flow highlights the translational nature of 3D printing research in thoracic surgery.

### 3.5 Authors

**Figure 6:**
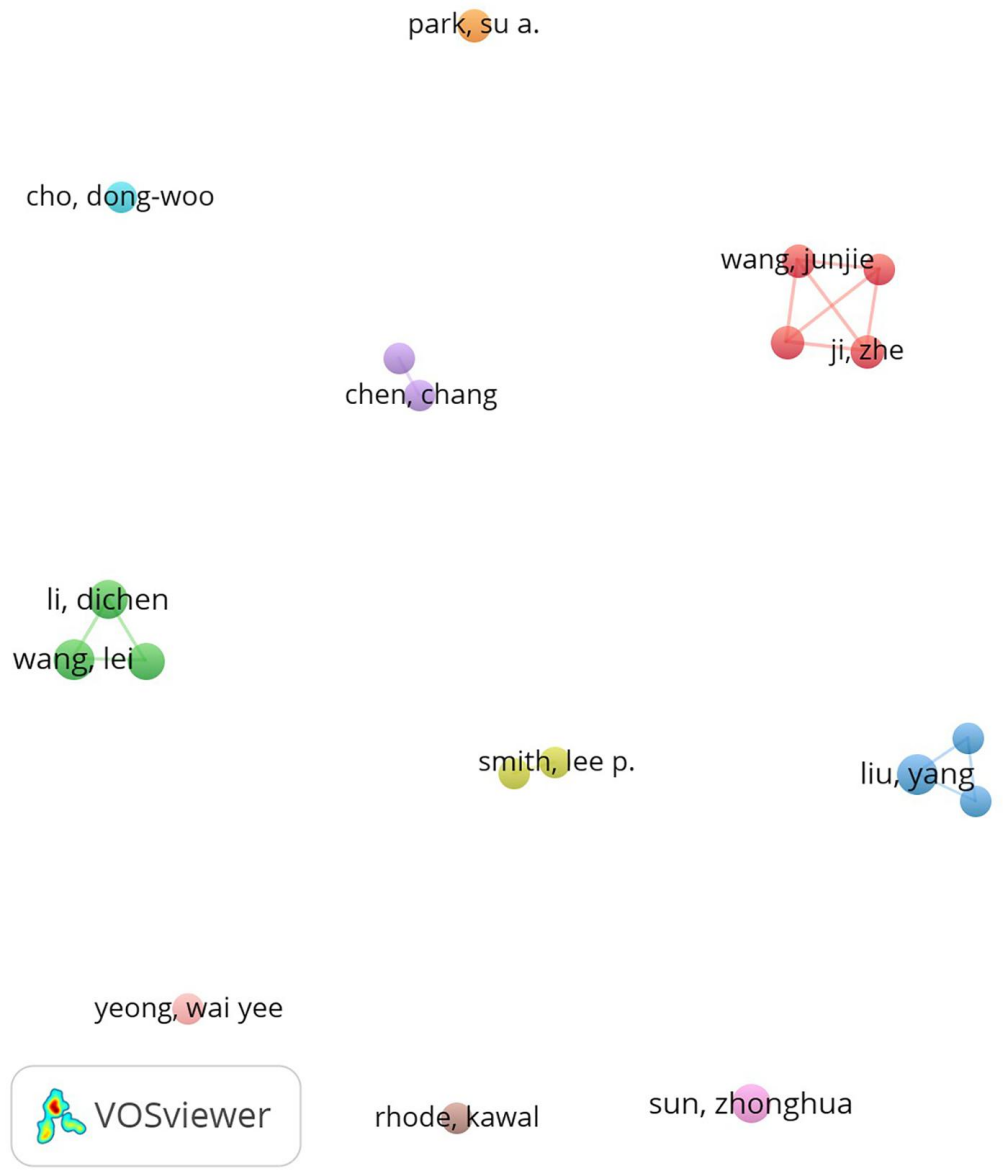
Cooperation network of authors.

Among all researchers who have published literature on 3D printing in thoracic surgery, the top 10 most prolific authors are listed in Table 5. Collectively, these authors contributed 80 publications, accounting for 10.81% of the total output in this field. Liu, Yang and Wang, Lei were the most productive authors, each publishing 10 papers, followed by Li, Dichen with 9 publications. The collaborative relationships among authors were visualized using CiteSpace (Figure 6), which illustrates the co-authorship network and highlights key nodes of collaboration and academic influence.

**Table 5:** Author's publications and co-citation table.

Rank	Author	Count	Rank	Co-cited author	Citation
1	Liu, yang	10	1	Park jh	45
2	Wang, lei	10	2	Morrison rj	44
3	Li, dichen	9	3	Zopf da	40
4	Sun, zhonghua	9	4	Wang l	36
5	He, jiankang	8	5	Murphy sv	34
6	Ji, zhe	7	6	Giannopoulos aa	29
7	Park, su a.	7	7	Valverde i	28
8	Sun, haitao	7	8	Biglino g	26
9	Wang, junjie	7	9	Rengier f	25
10	Chen, chang	6	10	Tino r	23

**Figure 7:**
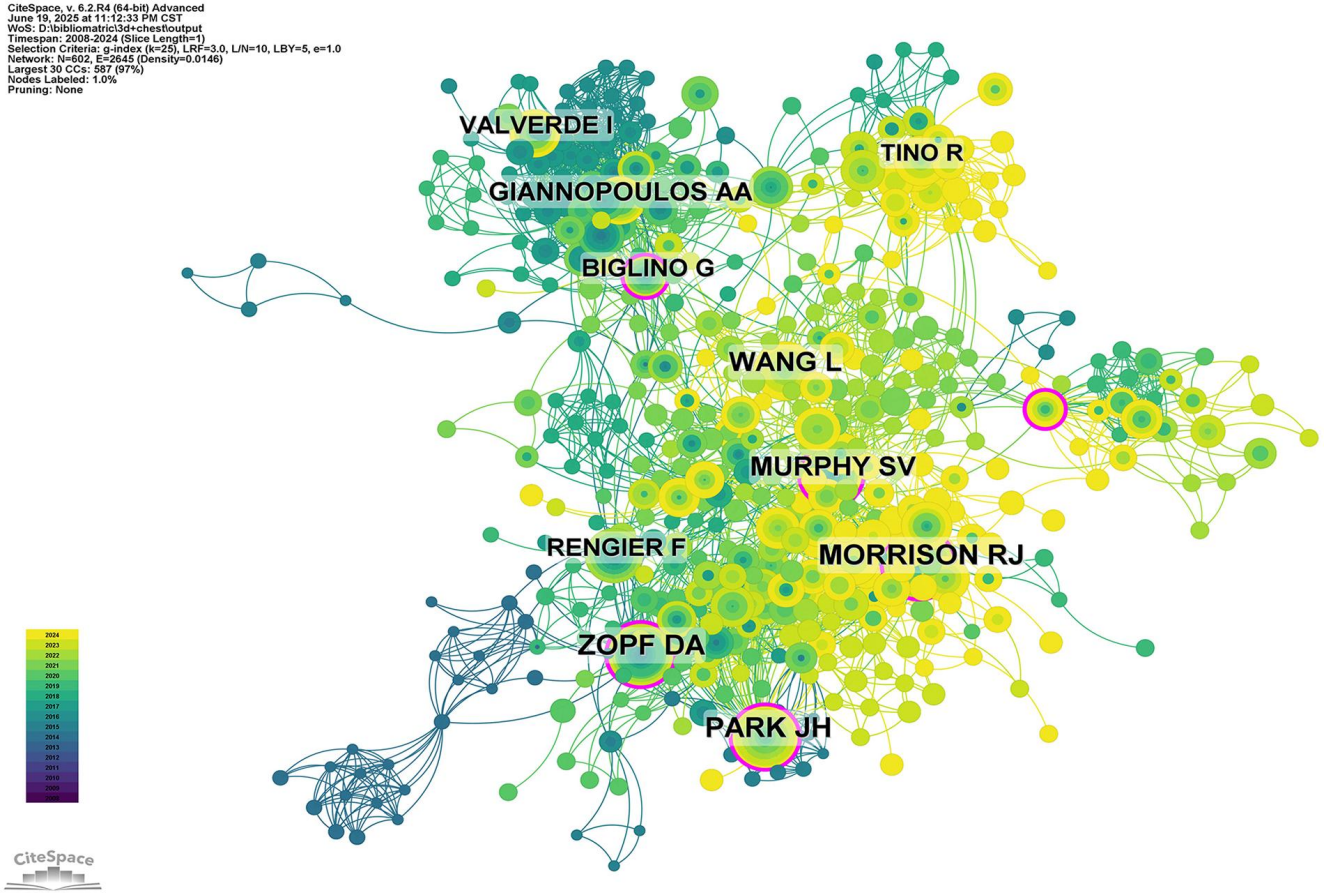
Co-citation network of authors.

Author impact was further assessed through co-citation analysis. Figure 7 and Table 5 present the top 10 most frequently cited and co-cited authors, respectively. A total of 14 authors were cited more than 20 times, indicating strong academic influence and visibility within the field. The largest nodes in the co-citation network corresponded to the most frequently co-cited authors, including Park JH (45 citations), Morrison RJ (44 citations), and Zopf DA (40 citations). These results underscore the foundational role of their research in shaping the academic discourse on 3D printing applications in thoracic surgery.

### 3.6 Citation and co-citation analysis

**Figure 8:**
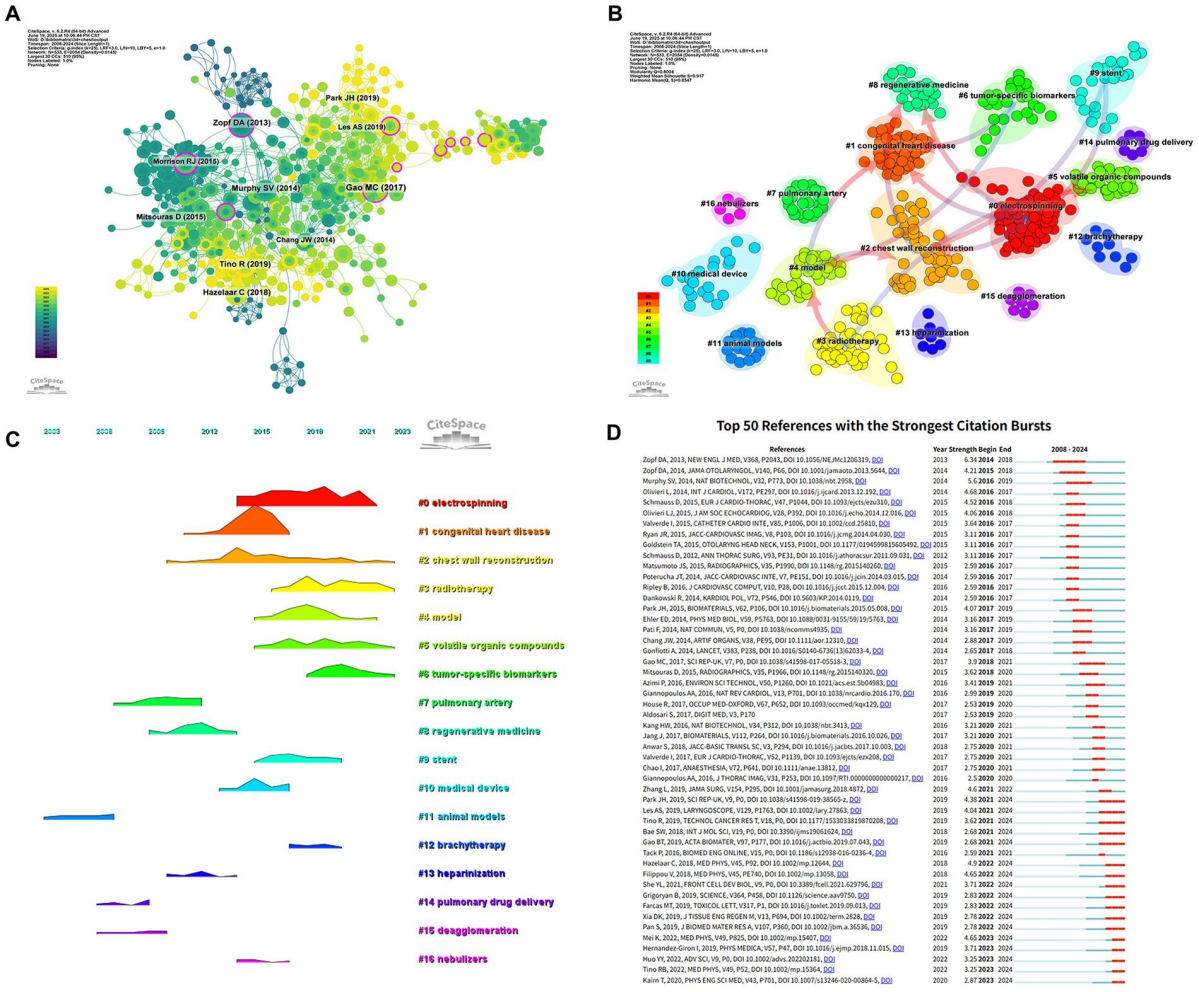
Analysis cited references and co-cited references. **(A)** Co-cited network of literature. **(B)** Clustering of co-cited literature. **(C)** Peak map of co-cited literature. **(D)** Bursting map of cited literature.

Using a one-year time slice and a time span from 2008 to 2024, a co-citation network of references was constructed, consisting of 533 nodes and 2,054 links (Figure 8A). Table 6 lists the top 10 most frequently co-cited references. The most highly co-cited article was published in Scientific Reports (UK), entitled “Tissue-engineered trachea from a 3D-printed scaffold enhances whole-segment tracheal repair”, with Gao MC as the first author. This study addressed the longstanding clinical challenge of long-segment tracheal stenosis and demonstrated the transformative potential of 3D printing technology in engineering functional tracheal replacements. The second most co-cited article was “A Systematic Review on 3D-Printed Imaging and Dosimetry Phantoms in Radiation Therapy”, which systematically reviewed the applications of additive manufacturing in radiation oncology, particularly emphasizing the use of 3D-printed phantoms for imaging quality evaluation and radiation dose measurement.

**Table 6:** Co-citation table of literature.

Rank	Title	Journal	Author(s)	Total citations
1	Tissue-engineered trachea from a 3D-printed scaffold enhances whole-segment tracheal repair	Sci Rep-UK	Gao MC	17
2	A Systematic Review on 3D-Printed Imaging and Dosimetry Phantoms in Radiation Therapy	Technol Cancer Res T	Tino R	16
3	Bioresorbable airway splint created with a three-dimensional printer	New Engl J Med	Zopf DA	16
4	3D bioprinting of tissues and organs	Nat Biotechnol	Murphy SV	15
5	Using 3D printing techniques to create an anthropomorphic thorax phantom for medical imaging purposes	Med Phys	Hazelaar C	15
6	Experimental Tracheal Replacement Using 3-dimensional Bioprinted Artificial Trachea with Autologous Epithelial Cells and Chondrocytes	Sci Rep-UK	Park JH	13
7	Medical 3D Printing for the Radiologist	Radiographics	Mitsouras D	13
8	Mitigation of tracheobronchomalacia with 3D-printed personalized medical devices in pediatric patients	Sci Transl Med	Morrison RJ	12
9	3D-printed, externally-implanted, bioresorbable airway splints for severe tracheobronchomalacia	Laryngoscope	Les AS	12
10	Tissue-engineered tracheal reconstruction using three-dimensionally printed artificial tracheal graft: preliminary report	Artif Organs	Chang JW	11

To further explore the knowledge structure and thematic evolution, we performed reference co-citation clustering and timeline analysis (Figures 8B,C). Distinct clusters revealed the temporal progression of research hotspots in this field. Early research foci included pulmonary artery (Cluster #7), animal models (#11), regenerative medicine (#8), heparinization (#13), pulmonary drug delivery (#14), and deagglomeration (#15). These clusters primarily reflected foundational research on biomaterials, preclinical models, and drug delivery platforms relevant to thoracic structures. Mid-phase research hotspots were represented by congenital heart disease (#1), medical devices (#10), and nebulizers (#16), indicating a transition toward applied clinical innovations and respiratory support technologies. Recent and ongoing frontiers were characterized by clusters such as electrospinning (#0), chest wall reconstruction (#2), radiotherapy (#3), model (#4), volatile organic compounds (#5), tumor-specific biomarkers (#6), stents (#9), and brachytherapy (#12). These clusters represent cutting-edge topics in biofabrication, precision oncology, imaging biomarkers, and thoracic surgical reconstruction—highlighting the multidisciplinary nature and translational orientation of current research.

In addition, we analyzed the top 354 keywords with the strongest citation bursts, which represent terms that have experienced a sharp increase in frequency over a specific period. We specifically focused on the top 50 keywords with the strongest burst strengths (Figure 8D). These keywords serve as indicators of emerging research hotspots and potential future directions in the field of 3D printing applications in thoracic surgery.

### 3.7 Keywords and hotspots

**Figure 9:**
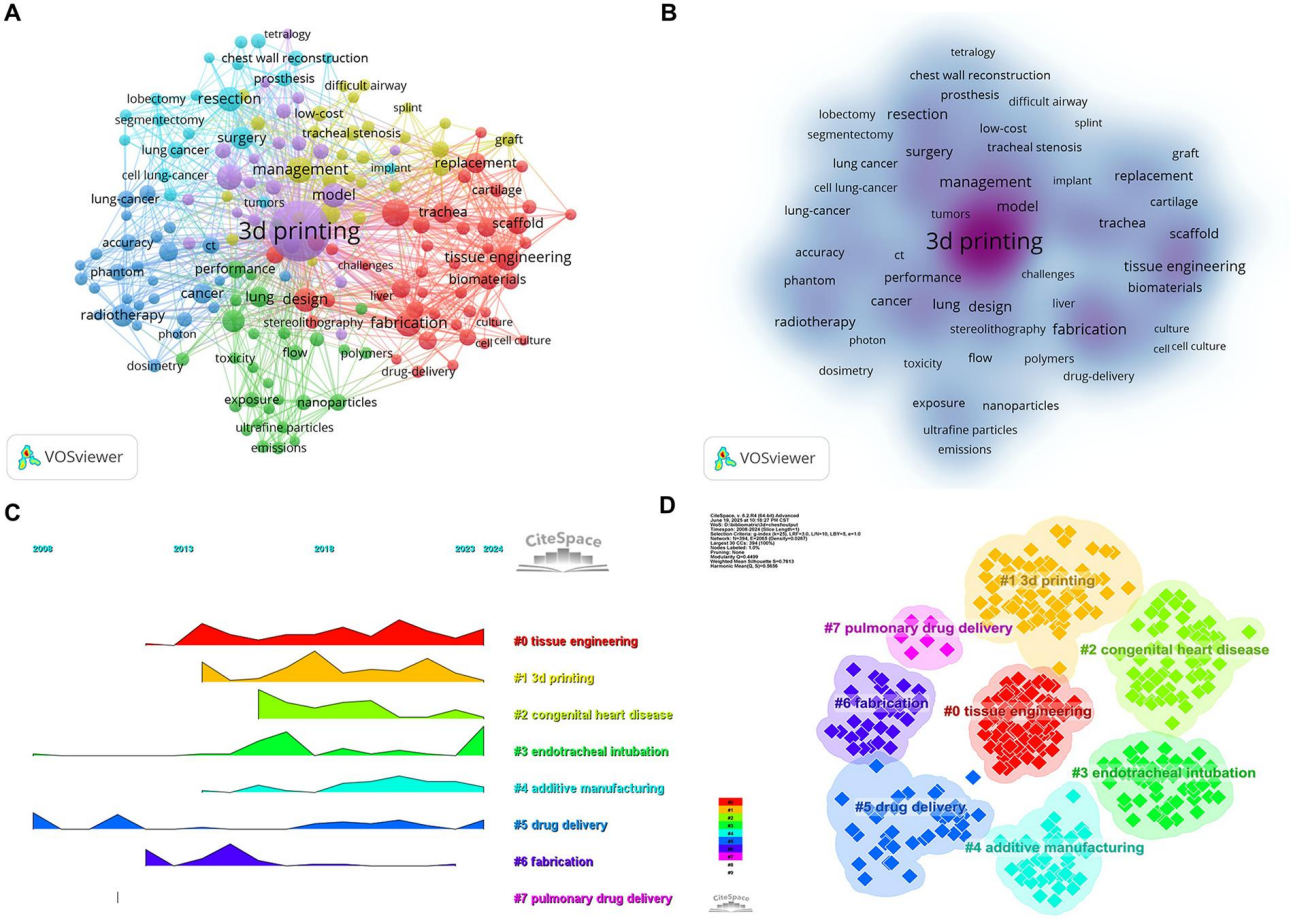
Analysis of keywords associated with 3D printing in thoracic disease. **(A)** Network map of high-frequency keywords. **(B)** Density map of keywords. **(C)** Peak map of keyword clustering. **(D)** Clustering map of keywords.

Keyword co-occurrence analysis using VOSviewer revealed that the most frequently used term was “3D printing” (290 occurrences), followed by “reconstruction” (47), “fabrication” (44), and “management” (42), as shown in Table 7 and Figures 9A,B. A volcano plot generated by CiteSpace (Figure 9C) illustrated the evolution of research hotspots over time, indicating that keywords such as “tissue engineering,” “congenital heart disease,” “endotracheal intubation,” “additive manufacturing,” and “drug delivery” have become prominent in recent years. After removing generic and non-informative keywords, a co-occurrence network of 173 keywords with a minimum occurrence threshold of six was constructed. These were grouped into six distinct clusters using VOSviewer (Figure 9D).

**Table 7:** High frequency keyword table.

Rank	Keyword	Counts	Rank	Keyword	Counts
1	3D printing	290	11	Radiotherapy	31
2	Reconstruction	47	12	Surgery	30
3	Fabrication	44	13	Scaffold	29
4	Management	42	14	Trachea	27
5	Tissue engineering	41	15	Cancer	25
6	Simulation	40	16	Lung	25
7	Model	38	17	Replacement	25
8	Resection	37	18	Children	24
9	Design	35	19	Biomaterials	22
10	Additive manufacturing	32	20	Computed tomography	22

**Figure 10:**
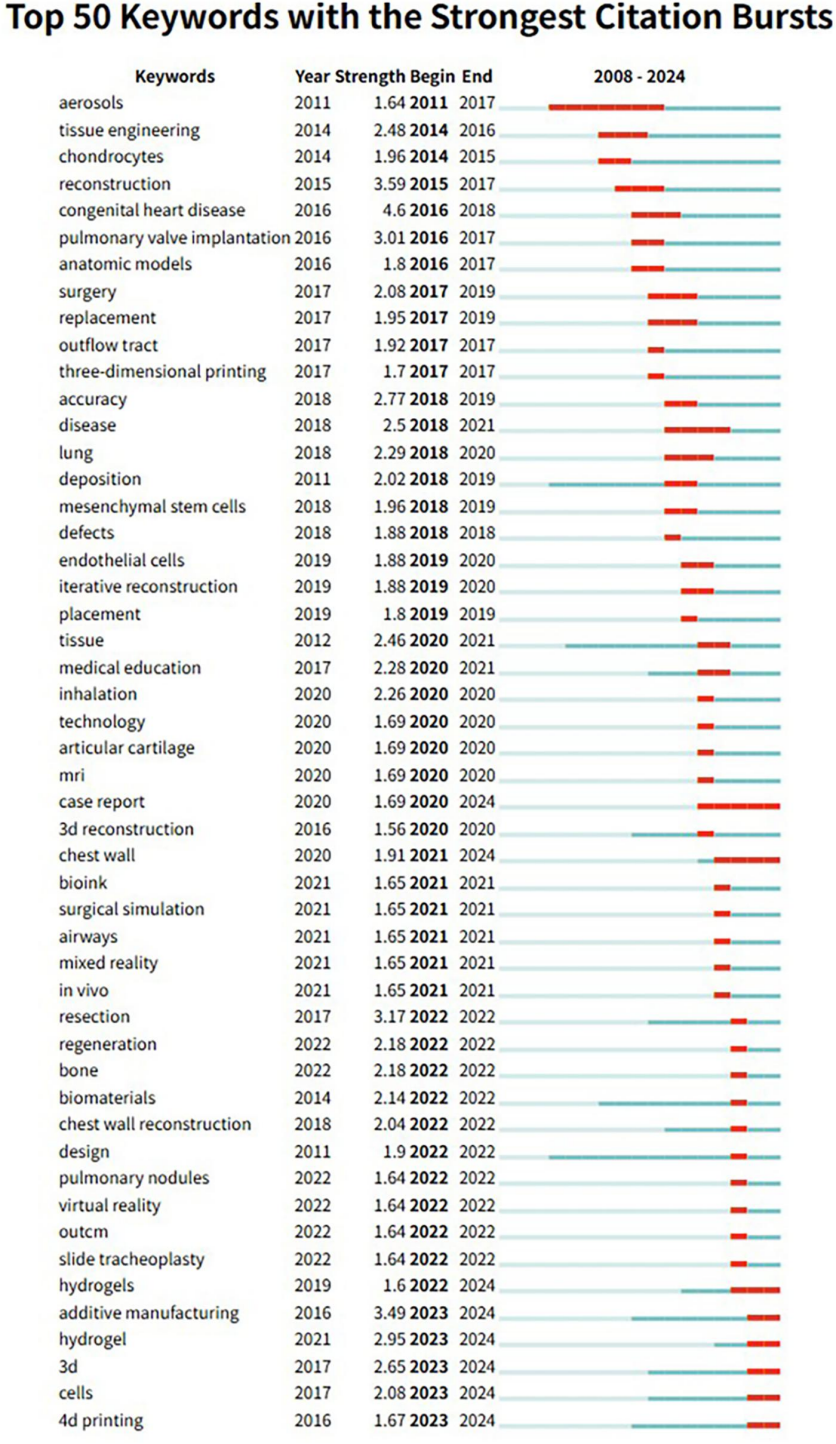
Bursting map of keywords.

Additionally, CiteSpace identified the 50 references with the strongest citation bursts between 2008 and 2024 (Figure 10), highlighting their significant influence. The most notable was an article published in The New England Journal of Medicine titled “Bioresorbable Airway Splint Created with a Three-Dimensional Printer” which demonstrated how high-resolution imaging and 3D bioprinting could enable patient-specific airway implants. Notably, 17 of the 50 burst references remain in an active citation phase, suggesting that research in 3D printing for thoracic surgery continues to attract considerable attention and is likely to remain a key focus in the coming years.

Our analysis of institutional centrality in the co-authorship network reveals a pattern of localized research efforts, particularly within countries like China, where high output is concentrated in domestic institutions. Although China has a large number of publications, the centrality of institutions in the global collaboration network is relatively low, indicating that much of the research remains isolated within national borders. In contrast, the United States, while not leading in publication volume, exhibits a much higher centrality value, suggesting that U.S. institutions are at the center of a broader, more integrated global research network. This pattern suggests that international collaboration is still limited, particularly in high-output countries like China, where there is a strong emphasis on domestic research. This finding highlights a key opportunity for improvement: increased interdisciplinary and international collaboration could accelerate the clinical translation of 3D bioprinting technologies and promote more robust global partnerships, facilitating faster innovation and cross-pollination of ideas.

## 4 Discussion

3D printing and bioprinting technologies are reshaping the landscape of thoracic surgery by enabling personalized, precise, and tissue-compatible solutions to some of the most challenging clinical problems in chest wall reconstruction, airway repair, and oncologic surgery. Our bibliometric analysis confirms not only a rapid increase in global publications over the past decade but also a notable shift in research focus—from early explorations of anatomical modeling and prosthetic simulation to the development of functional, bioactive, and even living tissue constructs through bioprinting. This transition reflects both technological maturation and increasing demand for personalized regenerative solutions in thoracic care.

Our co-citation analysis revealed distinct research clusters that highlight the progression of 3D printing in thoracic surgery. In the early years (2008–2015), research primarily focused on anatomical modeling and prosthetic design. This is evident from the clustering of keywords such as “surgical simulation” and “prosthetic implants,” which formed the core of early studies. However, by 2016, a transition occurred toward bioprinting and tissue engineering applications, as seen in the emergence of clusters related to “airway repair,” “lung reconstruction,” and “bioactive scaffolds.” This shift is consistent with the growing recognition of the potential for 3D printing to not only model anatomical structures but also fabricate biologically functional constructs. In recent years (2021–2024), research has further expanded into precision oncology, with a focus on tumor-specific implants and radiotherapy phantoms, reflecting the increasing importance of personalized treatment in thoracic surgery.

The collaboration network analysis further underscores the global nature of this field. China and the United States were identified as the two most productive countries, with strong bilateral cooperation. However, our analysis also revealed that, despite this prolific output, there are gaps in international collaboration, particularly between institutions in high-output countries. For example, while China has a leading publication volume, the United States has a higher citation impact, indicating the need for greater interdisciplinary partnerships to leverage the strengths of both regions. The collaboration networks highlight the importance of fostering closer ties between leading institutions in bioengineering, thoracic surgery, and materials science to accelerate the clinical translation of 3D bioprinting technologies.

The analysis of citation bursts revealed several research topics gaining significant attention in recent years. Notably, bioprinting and tissue engineering have seen substantial bursts in citations, reflecting the growing interest in creating functional, regenerative constructs for thoracic surgery. A particularly notable burst occurred in research related to bioresorbable airway splints and personalized airway implants, which have become prominent areas of study due to their potential to address complex conditions such as long-segment tracheal stenosis. These citation bursts indicate that the scientific community is increasingly focusing on the biologically integrated, regenerative potential of 3D printing in thoracic surgery, moving beyond traditional mechanical solutions.

Bioprinting, as a more advanced form of 3D printing, involves the layer-by-layer deposition of bioinks containing cells, extracellular matrix components, and growth factors to recreate complex tissue microenvironments (17, 18). In thoracic surgery, this approach has shown remarkable promise for airway, tracheal, and even lung tissue engineering (19). Unlike conventional 3D printing of inert materials, bioprinting allows for the generation of living constructs that can integrate with host tissue, promote healing, and potentially restore biological function (20). Several preclinical studies have demonstrated the feasibility of printing biocompatible tracheal scaffolds using hydrogels laden with chondrocytes or stem cells, which maintain structural integrity and support epithelialization post-implantation (21, 22). These advances are particularly relevant for conditions such as long-segment tracheal stenosis or pediatric airway malformations, where conventional grafts are limited by size mismatch, rigidity, and risk of infection.

Our analysis revealed that chest wall reconstruction remains one of the most extensively studied clinical applications of 3D printing in thoracic surgery (23). Customized prostheses made of titanium alloy or high-performance polymers have been used to replace resected ribs or sternum in tumor surgery and trauma, restoring both mechanical stability and cosmetic symmetry (24). However, such implants often lack biological integration. Bioprinting offers a future avenue to generate osteochondral or soft-tissue constructs tailored not only to anatomical dimensions but also to mechanical properties and cellular composition (25). For instance, hybrid printing strategies that combine structural polymers with cell-laden hydrogels can yield composite grafts suitable for load-bearing chest wall repair while facilitating tissue regeneration (26). Another emerging direction highlighted in our keyword burst and cluster analyses is the application of 3D printing and bioprinting in the creation of radiotherapy phantoms and tumor-mimetic models (27). These constructs enable personalized radiation dose planning, real-time procedural simulation, and *in vitro* drug screening. Tumor-specific 3D-printed models can replicate heterogeneous density and geometry of malignancies in the lung or mediastinum, thus enhancing both oncologic precision and training opportunities. Furthermore, biofabricated tumor models seeded with patient-derived cancer cells are now being developed as platforms for personalized medicine, bridging the gap between diagnostics and therapeutics (28, 29).

The development of bioinks is a critical component in advancing bioprinting applications in thoracic surgery (18). Current research is exploring materials that balance printability, mechanical stability, and biocompatibility—such as gelatin-methacrylate (GelMA), alginate, decellularized extracellular matrix (dECM), and fibrin-based hydrogels (30, 31). Incorporating tissue-specific dECM into bioinks enhances the physiological relevance of printed constructs, while functionalization with growth factors or microRNAs can further promote vascularization and immune tolerance post-implantation (32). For thoracic tissues exposed to dynamic respiratory motion and immunological stress, these innovations are essential to ensure long-term graft survival and integration. Despite these advances, significant translational challenges remain. One major limitation is the vascularization of thick or complex bioprinted tissues, which is critical for survival after implantation in highly perfused regions such as the lung. Novel strategies, including sacrificial ink printing for microchannel networks or incorporation of angiogenic cell types, are being actively investigated (33–35). Another bottleneck is regulatory approval, as few bioprinted constructs have progressed to clinical trials due to concerns over reproducibility, sterility, and long-term safety (36). Standardization of printing protocols, quality control, and bioink formulations will be crucial to ensure regulatory compliance and clinical adoption.

3D printing technology has shown tremendous potential in thoracic surgery, particularly in enhancing surgical precision, personalizing treatment, and improving patient outcomes. By utilizing patient-specific 3D-printed models, surgeons can gain a clearer understanding of complex anatomical structures, especially in the management of challenging conditions such as tumor resection, airway repair, and chest wall reconstruction. Personalized 3D models and implants help improve surgical visualization and safety, playing an essential role in preoperative planning, intraoperative navigation, and postoperative assessment. For example, 3D-printed airway stents and chest wall reconstruction implants have shown significant clinical efficacy in managing conditions such as tracheal stenosis and rib defects. The ability to match precise models to the patient's anatomy reduces surgical risks and accelerates recovery. Additionally, 3D printing has an essential role in tissue engineering, especially in thoracic surgery. Through bioprinting, which combines cells, scaffolding materials, and growth factors, biologically functional tissue constructs can be printed. In thoracic surgery, 3D bioprinting has the potential to create implants compatible with the patient's tissue, such as tracheal scaffolds that support epithelialization and tissue regeneration. As technology evolves, 3D printing is not only capable of producing structurally supportive models but also biologically active constructs, advancing the field of regenerative medicine. Furthermore, 3D printing in thoracic surgery is also pivotal in precision surgery and personalized medicine. In tumor resection surgeries, patient-specific 3D models, generated from CT or MRI data, allow surgeons to plan resections with greater accuracy, enhancing surgical precision and reducing intraoperative bleeding and complications (37). This personalized approach ensures better post-operative outcomes, particularly for complex cases involving tumor removal, airway reconstruction, and chest wall repair. Such personalized treatments are critical for patients with unique anatomical challenges or those requiring complex surgical interventions.

However, despite the vast potential of 3D printing technology in thoracic surgery, there are still several challenges to its clinical implementation. The primary obstacle is the high cost of 3D printing equipment and bioprinting materials. Currently, the cost of 3D printers and the associated maintenance is relatively high, especially for bioprinting, which requires specialized equipment and bioinks, along with cell-based materials that can be expensive. This financial barrier limits the widespread adoption of 3D printing, particularly in resource-limited settings or smaller medical institutions. Additionally, the process of creating high-fidelity, patient-specific models and implants requires specialized technical expertise, which may not be readily available in all hospitals. As a result, 3D printing technology is mostly used in advanced medical centers and research institutions, whereas its application in less resourceful regions remains limited. Another significant challenge lies in the biological compatibility and long-term performance of 3D-printed implants. While personalized implants made from 3D printing are tailored to a patient's anatomy and can be effective during surgery, their long-term biocompatibility and mechanical integrity still require thorough validation. For instance, in chest wall reconstruction, 3D-printed implants must withstand the mechanical stresses of the chest cavity, which may lead to implant failure if the material lacks sufficient strength. Additionally, the biological degradation of these implants, tissue integration, and cellular adhesion need further research. Many 3D-printed implants are still in the clinical trial phase, and there is a lack of long-term clinical data to confirm their safety and effectiveness. Furthermore, regulatory and standardization challenges remain. While 3D printing technologies have made significant progress in various applications, there is a lack of established regulatory frameworks specifically for 3D-printed medical devices. Compared to traditional medical devices and pharmaceuticals, 3D-printed implants often lack stringent regulatory standards, which could lead to substandard products entering the market. The use of biological materials and cell-based bioprinting raises additional ethical concerns and complicates the approval process. Ensuring that biofabricated products meet safety, reproducibility, and quality standards is critical for the widespread clinical adoption of 3D printing in thoracic surgery. In addition to these technological and regulatory challenges, the implementation of 3D printing in thoracic surgery requires multi-disciplinary collaboration. Although 3D printing has enormous potential, its successful application requires the collaboration of thoracic surgeons, engineers, material scientists, biologists, and regulatory bodies. Strengthening cross-disciplinary cooperation and promoting the standardization and regulation of 3D printing technologies are key to accelerating the clinical translation of these innovations. Furthermore, multi-center clinical trials and long-term follow-up studies are necessary to establish the clinical benefits and safety profiles of 3D-printed implants in thoracic surgery.

Despite these challenges, the future of 3D printing in thoracic surgery remains promising. As technology advances and interdisciplinary collaborations grow, 3D printing is expected to play a transformative role in personalized treatment, tissue engineering, and precision medicine in thoracic surgery. Looking ahead, the integration of 3D bioprinting with advanced imaging, artificial intelligence, and robot-assisted surgery may further enhance its clinical utility (38). Robotics may facilitate minimally invasive implantation of bioengineered constructs with sub-millimeter precision (39). Additionally, combining bioprinting with organ-on-a-chip platforms may enable dynamic simulation of pulmonary physiology or tumor–stroma interactions *in vitro*, opening new possibilities in translational research and drug development (40).

## 5 Conclusion

This study reveals the rapid evolution of 3D printing, particularly bioprinting, in thoracic surgery, as evidenced by the steady increase in publications from 2021 to 2024. Early focus on anatomical modeling and prosthetic design has shifted toward biofabrication of functional tissue constructs. Our analysis highlights key trends, including the integration of bioprinting with advanced imaging and AI, as well as a growing emphasis on tissue engineering for airway and chest wall reconstruction. However, challenges remain in vascularization, regulatory approval, and global collaboration. Addressing these issues will be crucial to realizing the full potential of bioprinting, which is poised to transform thoracic surgery by combining personalized design with regenerative medicine.

## Data Availability

The original contributions presented in the study are included in the article/Supplementary Material, further inquiries can be directed to the corresponding author.
